# Kimura’s disease: An uncommon cause of lymphadenopathy

**DOI:** 10.4103/0971-5851.73598

**Published:** 2010

**Authors:** Veerendra Kumar, Salini, Savida Haridas

**Affiliations:** *Department of Pediatrics, Institute of Child Health, Government Medical College, Kottayam, Kerala, India*

**Keywords:** *Angiolymphoid hyperplasia*, *eosinophilic abscess*, *eosinophilic granuloma*, *glomerulonephritis*, *hypertrophy*, *Kimura*, *salivary gland*, *thrombotic storm*

## Abstract

Lymph node enlargement of neck and axilla is one of the common presenting complaints in pediatrics. We are presenting here a very rare cause of axillary lymphadenopathy detected in a toddler.

## INTRODUCTION

Kimura’s disease is a rare entity causing subcutaneous swellings and lymphadenopathy, with hardly 120 cases reported world wide.[1] It is mainly seen in Asian countries. We are reporting the case of a 3-year old child affected by this rare disease. Even though it is a benign disease in a majority of cases, it can produce devastating renal and thrombotic complications.[2] Therefore, it needs proper follow-up.

## CASE REPORT

A 3-year old male child presented to us with swelling in the left axilla of 1.5 months duration. He had no history of prolonged fever, cough, fatigue, loss of weight, contact with tuberculosis, bleeding manifestations, or bone and joint pains. On examination, he was afebrile, with normal growth, no pallor or clubbing. In the left axilla, there were two mobile, nontender, lymph nodes of sizes 4×3 and 3×2 cms with normal skin over them. Other groups of lymph nodes were not enlarged. No hepatosplenomegaly was seen.

His blood counts showed eosinophilia (40%); peripheral smear was within normal Limits, except for eosinophilia. Chest X-ray and skeletal surveys were normal. Gastric aspirate was negative for AFB. Skin tuberculin test was negative. Urine and stool analyses did not show any abnormality. Serum Ig E was 1026 IU/ml (<180 IU/ml).

The swellings were excised and sent for histopathological examination. Examination of the excised swelling showed lymph node architecture with marked hyperplasias of germinal centers. Extensive infiltration by mature eosinophils was present with occasional areas of eosinophilic abscess. In paracortical region, hyalinized vessels were seen.

**Figure 1:**
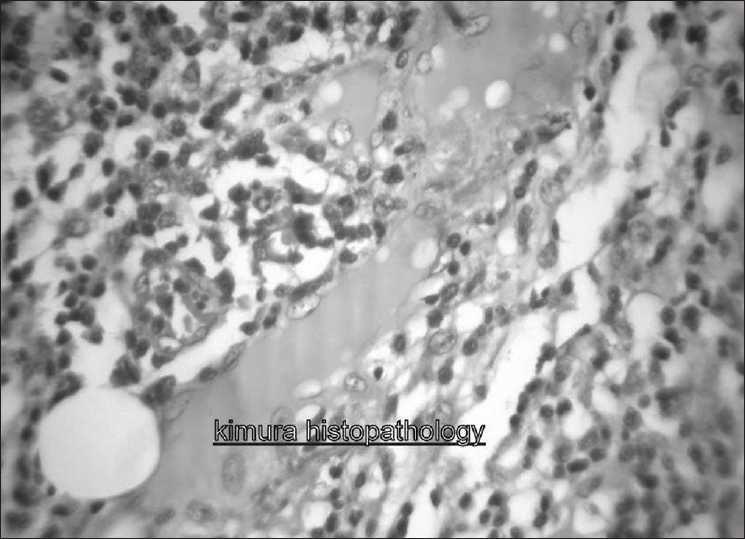
Histologic examination of biopsy specimen showing extensive infiltration by mature eosinophils and eosinophilic abscess. Plasma cells and mast cells also seen

Sinusal and paracortical sclerosis was present. Plenty of plasma cells and mast cells were also seen [Figure 1]. CD1A marker was negative on immunohistochemistry (positive result is an indicator of langerhan cell histiocytosis). After excision, he was advised to come for regular follow up. He has not so far developed any new lesions or complications.

## DISCUSSION

This picture is suggestive of Kimura’s disease which usually presents as painless, sometimes disfiguring, subcutaneous nodules in the head and neck region, with eosinophilia and adenopathy.[1] Although, it may mimic a neoplastic process, it is a chronic inflammatory disease of unknown cause. The disease progresses slowly and is endemic to Asians. Kimura’s disease was first described in China in 1937, but it was not referred to as “Kimura’s disease” until its description in the Japanese language literature in 1948.[1]

Common sites of involvement are the parotid glands and the epitrochlear, axillary, and inguinal nodes. Although the masses enlarge slowly, patients remain asymptomatic otherwise. Pruritus and dermatitis may occur, and rare sites of involvement include the kidneys, orbits, ears, spermatic cord, and nerves. Common type of renal involvement is nephrotic syndrome.[1,3] Widespread disseminated intravascular thrombosis is also reported in literature, affecting mesenteric and renal veins (thrombotic storm).[3]

Etiology is thought to be an abnormal immune response to an unknown antigenic stimulus.

The constant histologic features which are seen in this disease are preserved lymph node architecture, florid germinal centers, eosinophilic infiltration, and an increased amount of postcapillary venules. The frequent features include sclerosis, karyocytosis in both the germinal centers and the paracortex, vascularization of the germinal centers, proteinaceous deposits in germinal centers, necrosis of germinal centers, eosinophilic abscesses, and atrophic venules in sclerotic areas.[1,4]

The aforementioned histologic findings differentiate Kimura’s disease from more common causes of head and neck masses. Malignancy should be ruled out first. The absence of Reed-Sternberg cells helps to exclude Hodgkin’s disease. Unfortunately, T cell lymphomas can present with polymorphonuclear lymphocytes and eosinophilia, making the distinction difficult.[1] Although atypical, histiocytosis-X can present with subcutaneous masses, the diagnosis is made by finding the characteristic abnormal histiocytes[1] and detecting CD1A marker.

Differentiating Kimura’s disease from angiolymphoid hyperplasia with eosinophilia requires a strict analysis of clinical and histologic features because the diseases are similar and were once thought to be the same disorder. Both the diseases usually present with soft tissue masses in the head and neck region, but in angiolymphoid hyperplasia with eosinophilia, the lesions are mostly dermal or subcutaneous and not often in lymph nodes, which is a common location for Kimura’s lesions. Angiolymphoid hyperplasia with eosinophilia is more typically seen in middle-aged women and Kimura’s disease in younger men.[1]

Although spontaneous resolution has been reported, most patients have a prolonged course with slow enlargement of the masses.[2] There is no potential that the lesions will become malignant.[2] Treatment of Kimura’s disease is not codified.[2] Surgical excision of lesion(s) is the first - line therapy even though relapses are frequent.[2] Systemic corticotherapy with prednisone is indicated for relapses and for renal involvement with good efficacy, but with a risk of relapse when the treatment is withdrawn.[2] Radiation treatment is usually used for the local control of lesions not responsive to steroids, and total doses of 20-30 Gy have proved effective. Irradiation should be considered not only in patients resistant to steroids but also in young patients in whom the long-term side effects of steroids may be more deleterious than a limited course of irradiation which may prevent relapse.[5]

Other treatment modalities are intralesional administration of steroids, cytotoxic agents, and electrodesiccation. All-trans-retinoic acid with low dose prednisone induced remission in one case. Pranlukast, a leukotrine receptor antagonist, and cetrizine, an H1 receptor blocker, were also effective in inducing clinical remission in a few cases.[2]
